# O03 The impact of COVID-19 infection prevention and control measures on transmission of hospital-acquired infections and antimicrobial resistance in Africa

**DOI:** 10.1093/jacamr/dlac003.002

**Published:** 2022-02-16

**Authors:** Linzy Elton, Muzamil M. Abdel Hamid, John Tembo, Hana Elbadawi, Kwitaka Maluzi, Mohamed H. Abdelraheem, Timothy D. McHugh, Isobella Honeyborne, Kerry Roulston, Margaret J. Thomason, Alimuddin Zumla

**Affiliations:** Centre for Clinical Microbiology, University College London, London, UK; Institute of Endemic Diseases, University of Khartoum, Khartoum, Sudan; HerpeZ, University Teaching Hospital, Lusaka, Zambia; Institute of Endemic Diseases, University of Khartoum, Khartoum, Sudan; HerpeZ, University Teaching Hospital, Lusaka, Zambia; Institute of Endemic Diseases, University of Khartoum, Khartoum, Sudan; Centre for Clinical Microbiology, University College London, London, UK; Centre for Clinical Microbiology, University College London, London, UK; Centre for Clinical Microbiology, University College London, London, UK; Centre for Clinical Microbiology, University College London, London, UK; Centre for Clinical Microbiology, University College London, London, UK; National Institute for Health Research, UK

## Abstract

**Background:**

Patients who develop serious illness due to COVID-19 are more likely to have bacterial coinfections, for which WHO recommends treatment with antibiotics. As a result, many countries are observing a change in antimicrobial stewardship (AMS), in addition to changes in infection prevention and control (IPC) practices such as the use of personal protective equipment, on COVID-19 wards. Few data on COVID-19 and its impact on nosocomial infections and antimicrobial resistance (AMR) are available from low and middle-income countries (LMICs). As these countries often have high rates of AMR, it is vital to report the effects of COVID-19 on AMS so as to inform clinical practice and IPC guidelines. This study aims to compare prevalence of AMR in COVID-19 wards with general non-COVID-19 hospital wards.

**Methods:**

This pilot hospital-based study is being conducted in two sites in both Sudan and Zambia. IPC and AMS guidelines for COVID-19 and non-COVID-19 wards were identified for each institution. This study is comparing bacterial isolates and AMR patterns of nosocomial associated infections acquired on COVID-19 and non-COVID-19 wards were compared, using microbiological and sequencing methods. A total of 200 patients have been recruited: 100 per country, 50 COVID-19 patients and 50 non-COVID-19 patients. AMR transmission patterns are being identified using Oxford Nanopore Technologies sequencing for phylogenetic analysis.

**Results:**

The study began recruiting in May 2021 and completed recruitment of patients in October 2021. The majority of microbiological laboratory work will be completed within Q3 2021, with analysis of the results and sequencing completed in Q4 2021. A half-way point summary analysis of the data suggests differences in patient profiles, both between COVID-19 and non-COVID-19 wards at both sites, as well as differences between the two countries. Preliminary analysis also suggests a significant difference between the prevalence of MDR infections in Gram-negatives seen between COVID-19 (53% in Sudan and 83% in Zambia) and non-COVID-19 (14% Sudan, 33% Zambia) (*t*-test, *P=*0.0032 Sudan, *P=*0.0455 Zambia) ward patients in both countries (see Figure 1).

**Conclusions:**

The study is providing evidence to inform policy on IPC and AMS measures to be implemented on COVID-19 wards. In addition, the outcomes of the study will be used to create a pragmatic sequencing pipeline for potential AMR outbreaks suitable for use in LMICs clinical settings.

**Figure 1.**
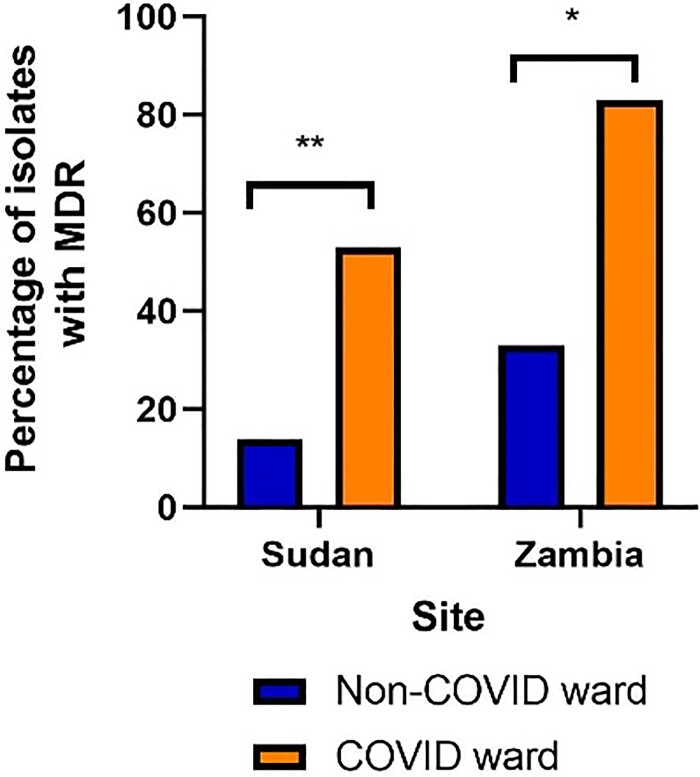
Percentage of Gram-negative bacteria isolated from patients on COVID-19 and non-COVID-19 wards in Sudan and Zambia, showing significant difference between the wards in both countries (*t*-test, *P* = 0.0032 Sudan, *P* = 0.0455 Zambia).

